# Supplementary material for: Molecular insights into degron recognition by CRL5ASB7 ubiquitin ligase
Source: Nat Commun. 2024 Jul 22;15:6177. doi: 10.1038/s41467-024-50556-x (PMC11263391; doi:10.1038/s41467-024-50556-x)
Supplement: Supplementary file 1 — Supplementary information [file 41467_2024_50556_MOESM1_ESM.pdf]

Supplementary information for

Molecular insights into degron recognition by $CRL5^{ASB7}$ ubiquitin ligase

Mengyu Zhou[1,#], Xiaolu Wang[2,#], Jiangtao Li[1], Jinfeng Ma[1], Ziyu Bao[3], Xiaojie Yan[1], Bing Zhang[1], Tong Liu[4], Ying Yu[2,*], Wenyi Mi[3,*] and Cheng Dong[1,4*]

[1]The Province and Ministry Co-sponsored Collaborative Innovation Center for Medical Epigenetics, Key Laboratory of Immune Microenvironment and Disease (Ministry of Education), Department of Biochemistry and Molecular Biology, School of Basic Medical Sciences, Tianjin Medical University, Tianjin, 300070, China

[2]Department of Pharmacology, Tianjin Key Laboratory of Inflammatory Biology, Center for Cardiovascular Diseases, Tianjin Medical University, Tianjin, 300070, China

[3]Tianjin Institute of Immunology, Department of Immunology, School of Basic Medical Sciences, Tianjin Medical University, Tianjin 300070, China

[4]Department of Cardiology, Tianjin Institute of Cardiology, Second Hospital of Tianjin Medical University, Tianjin, 300211, China

*Corresponding author. Email: dongcheng@tmu.edu.cn; wenyi.mi@tmu.edu.cn; yuying@tmu.edu.cn

[#]These authors contributed equally to this work.

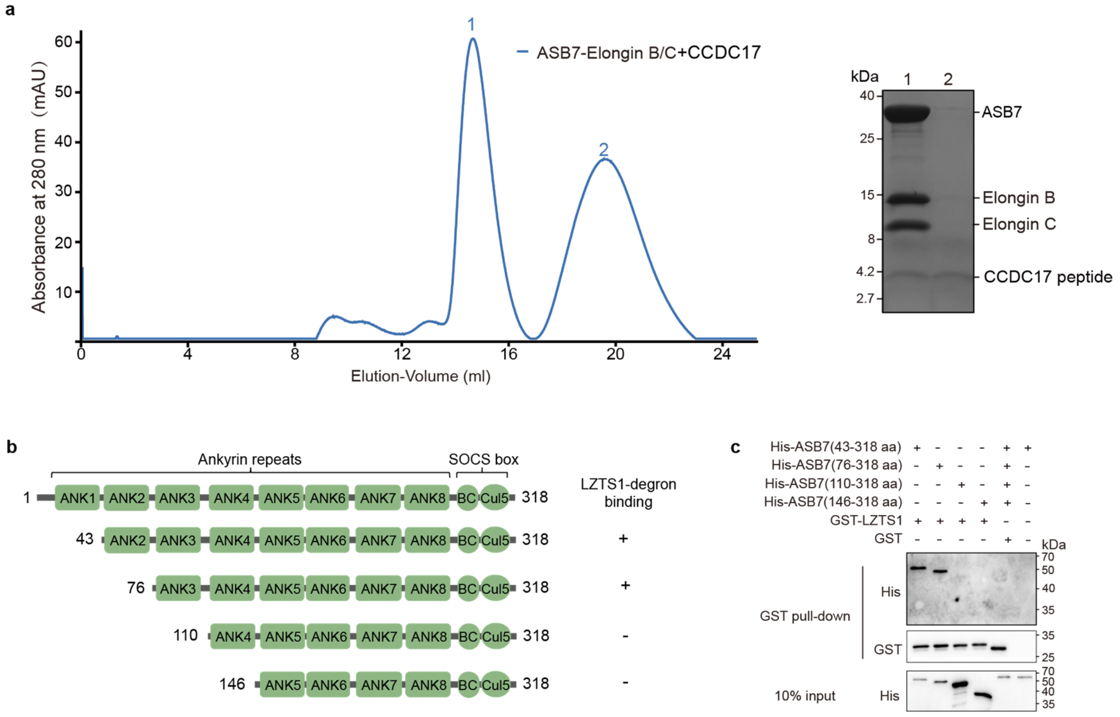

Supplementary Fig. 1 | ASB7 interacts with its cognate degron *in vitro*. a, Left, gel-filtration chromatography profile of ASB7 in complex with CCDC17-degron, as analyzed by Superdex 200 Increase 10/300 GL. Right, SDS-PAGE gel of the peak fractions, stained with Coomassie blue. Source data are provided as a Source Data file. Representative image, n=3. **b**, Domain architecture of human ASB7. ANK, ankyrin repeat; BC, Elongin BC-binding motif; Cul5, cullin 5-binding motif; SOCS, suppressors of cytokine signaling. **c,** Western blot analysis of GST pull-down assay using GST-fused LZTS1-degron as bait and purified different His-SUMO tagged ASB7 truncated mutants with Elongin B/C as prey. Source data are provided as a Source Data file. Representative image, n=3.

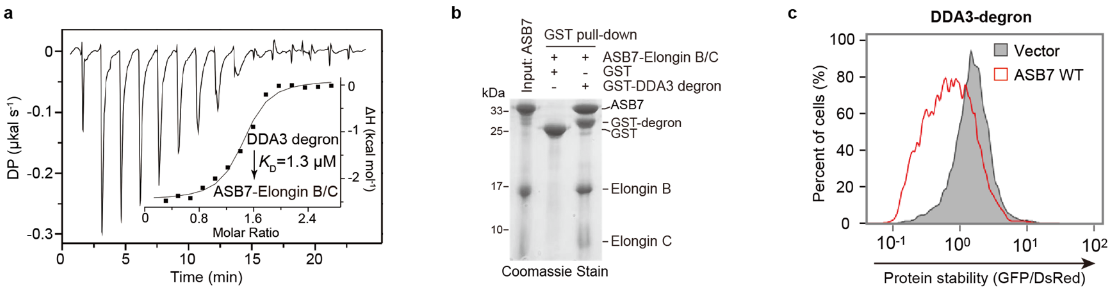

Supplementary Fig. 2 | ASB7 targets DDA3 for degradation. a, ITC measurement of binding affinity of DDA3 peptide to wild-type ASB7. Source data are provided as a Source Data file. **b**, GST pull-down assay using GST-fused DDA3-degron to pull down purified ASB7-Elongin B/C complex. Source data are provided as a Source Data file. Representative image, n=3. **c**, Stability analysis of GFP-fused DDA3-degron with overexpression of exogenous WT ASB7. The ratio of GFP/DsRed was analyzed by flow cytometry.

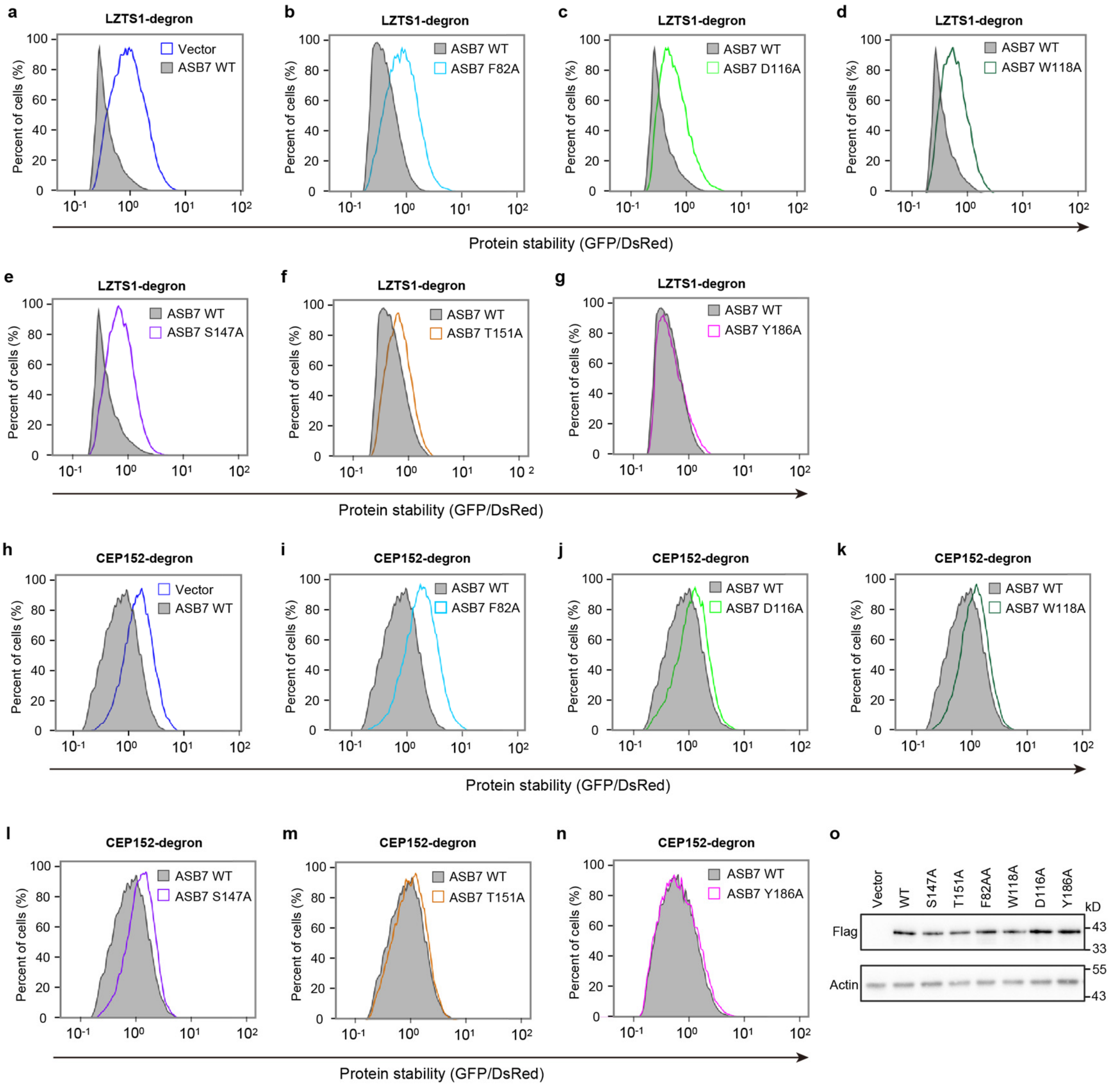

Supplementary Fig. 3 | GPS analysis of the key residues for degron degradation. a-g, Stability analysis of GFP-fused LZTS1-degron with overexpression of exogenous WT and mutant ASB7 in HEK293T cells. The GFP/DsRed ratio was analyzed by flow cytometry. **h-n**, Stability analysis of GFP-fused CEP152-degron with overexpression of exogenous WT and mutant ASB7. **o**, Western blot analysis of Flag-tagged WT and mutant ASB7 expression in GPS-reporter cell lines. Source data are provided as a Source Data file. Representative image, n=3.

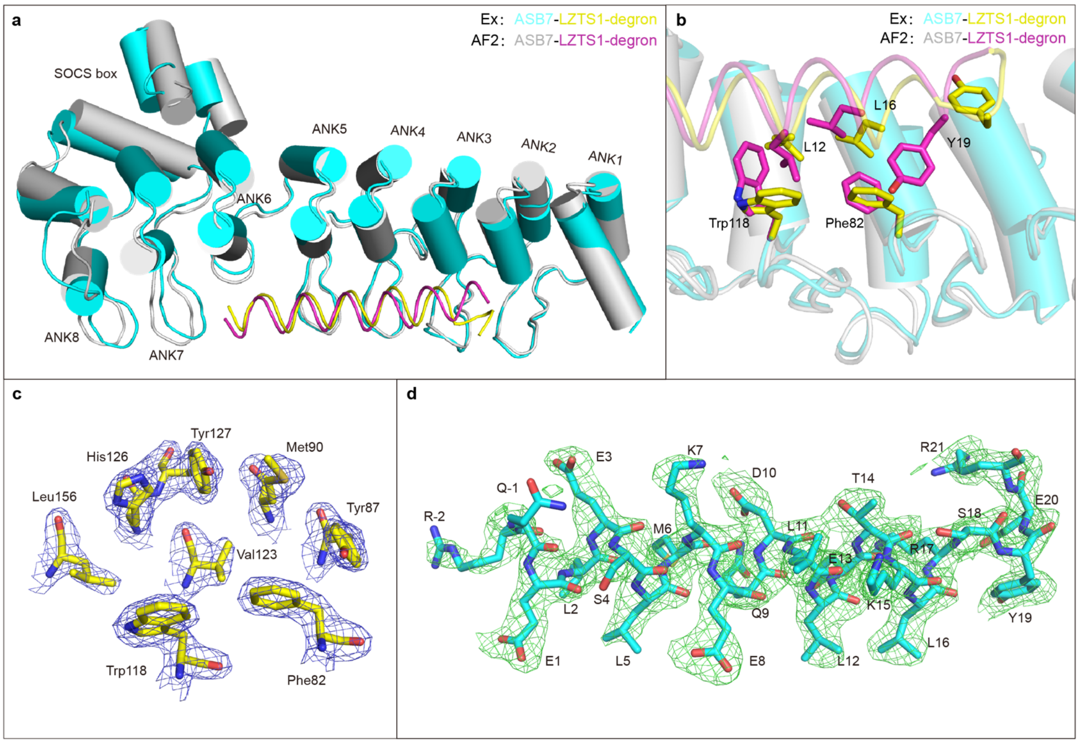

Supplementary Fig. 4 | Comparison of AlphaFold2 predicted model with experimentally determined structure. a, Superimposition of the AlphaFold2 predicted ASB7 bound degron model with our experimentally determined structure. **b,** The aromatic residues Trp118 and Phe82 of ASB7 are flipped by 90 degrees in the experimentally determined structure, and stack well with degron residues L12 and L16, respectively. The degron residue Y19 is flipped by ~180 degrees. **c,** 2Fo-Fc electron density map (blue mesh) of the ASB7 residues surrounding substrate residues L12 and L16, countered at the 2.0 σ level. **d,** Fo-Fc omit map (green mesh) of the LZTS1-degron, countered at the 2.0 σ level.

For Fig.5
LZTS1

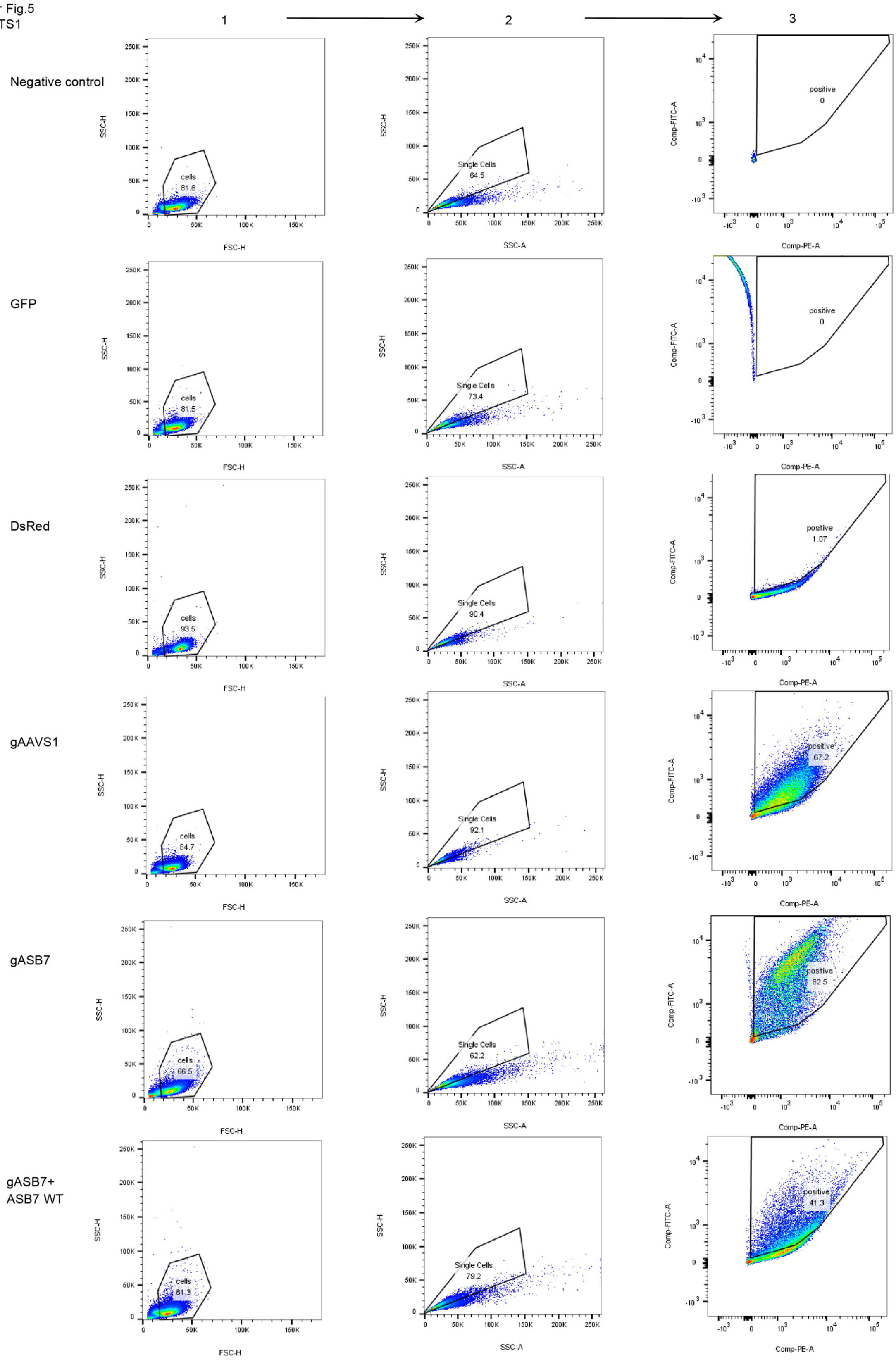

For Fig.5
LZTS1

gASB7+
ASB7 F82A

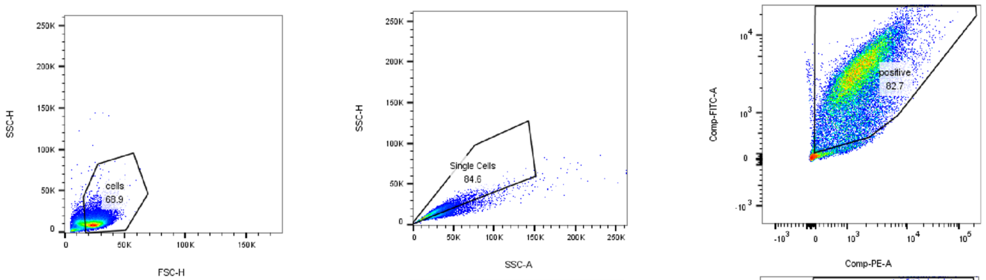

gASB7+
ASB7 D116A

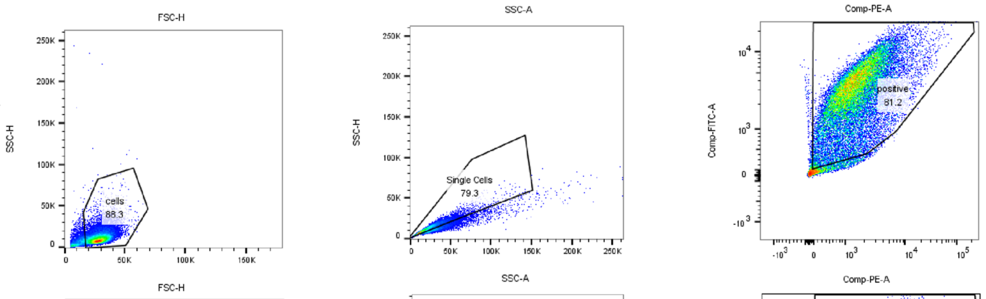

gASB7+
ASB7 W118A

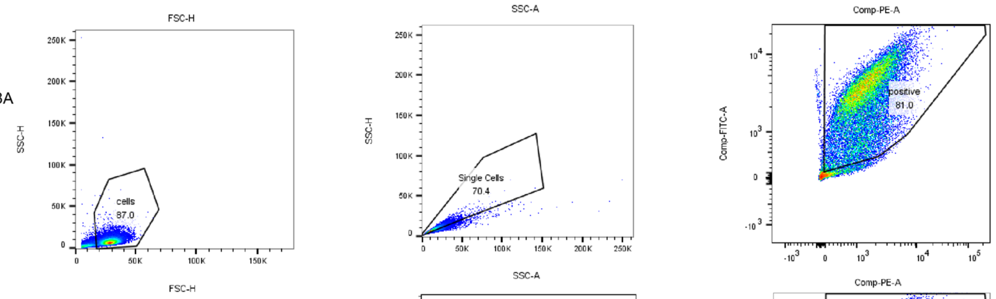

gASB7+
ASB7 S147A

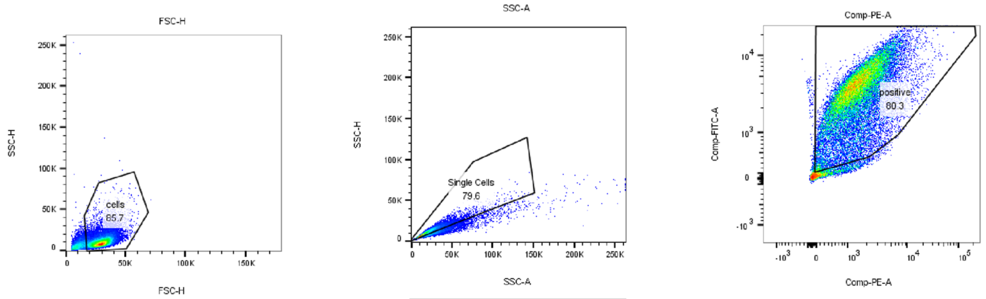

gASB7+
ASB7 T151A

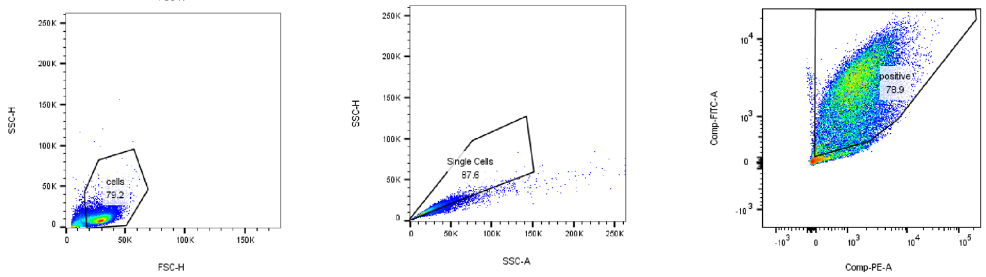

For Fig.5
LZTS1

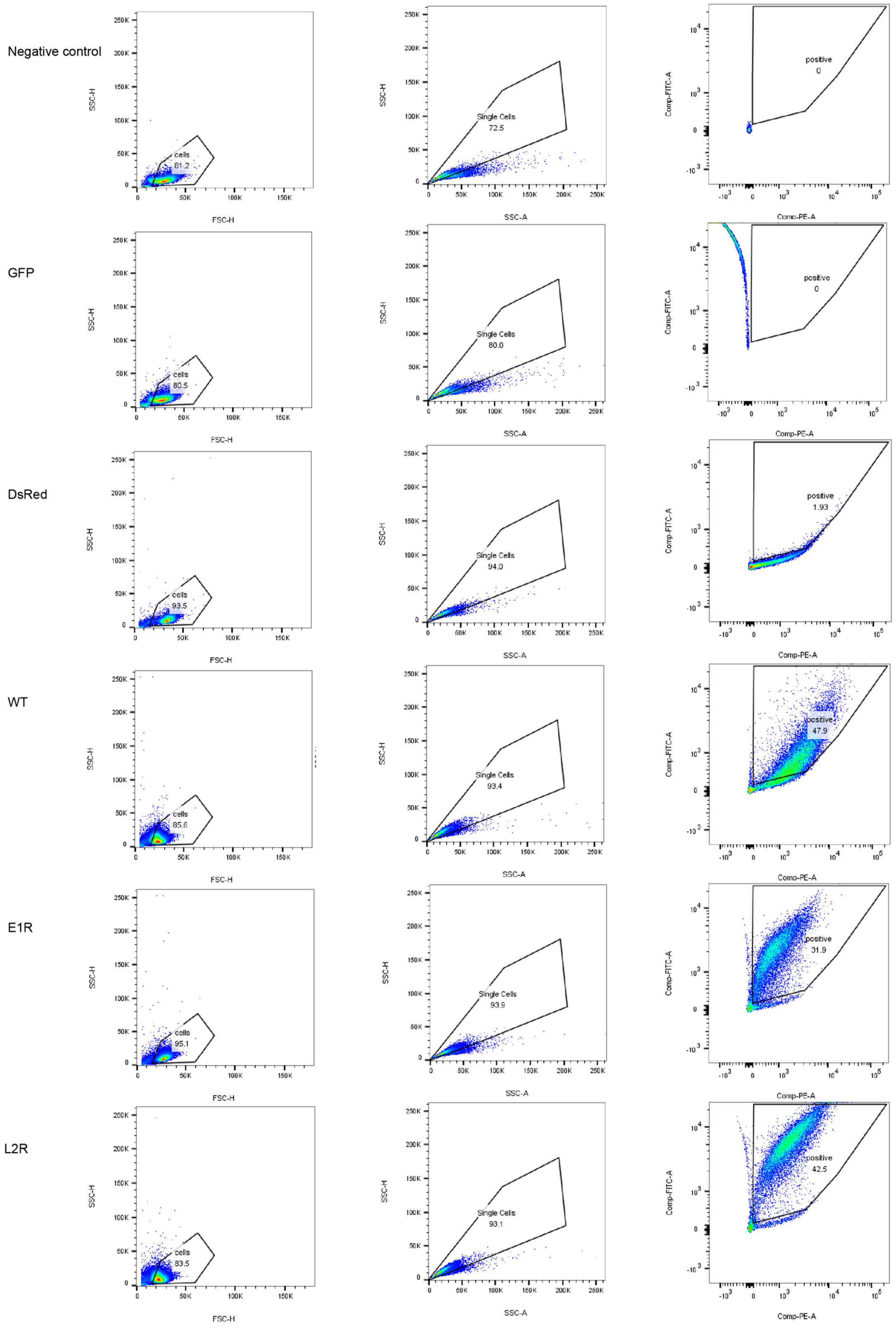

For Fig.5
LZTS1

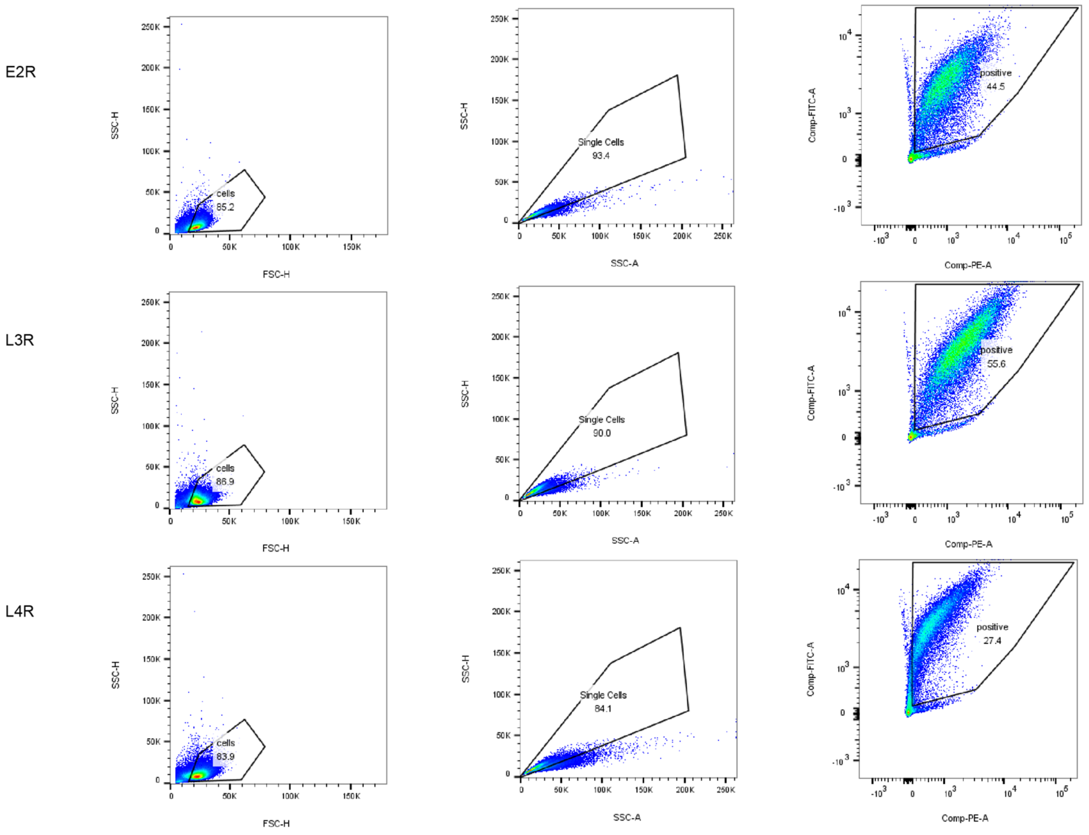

For Fig.5
CEP152

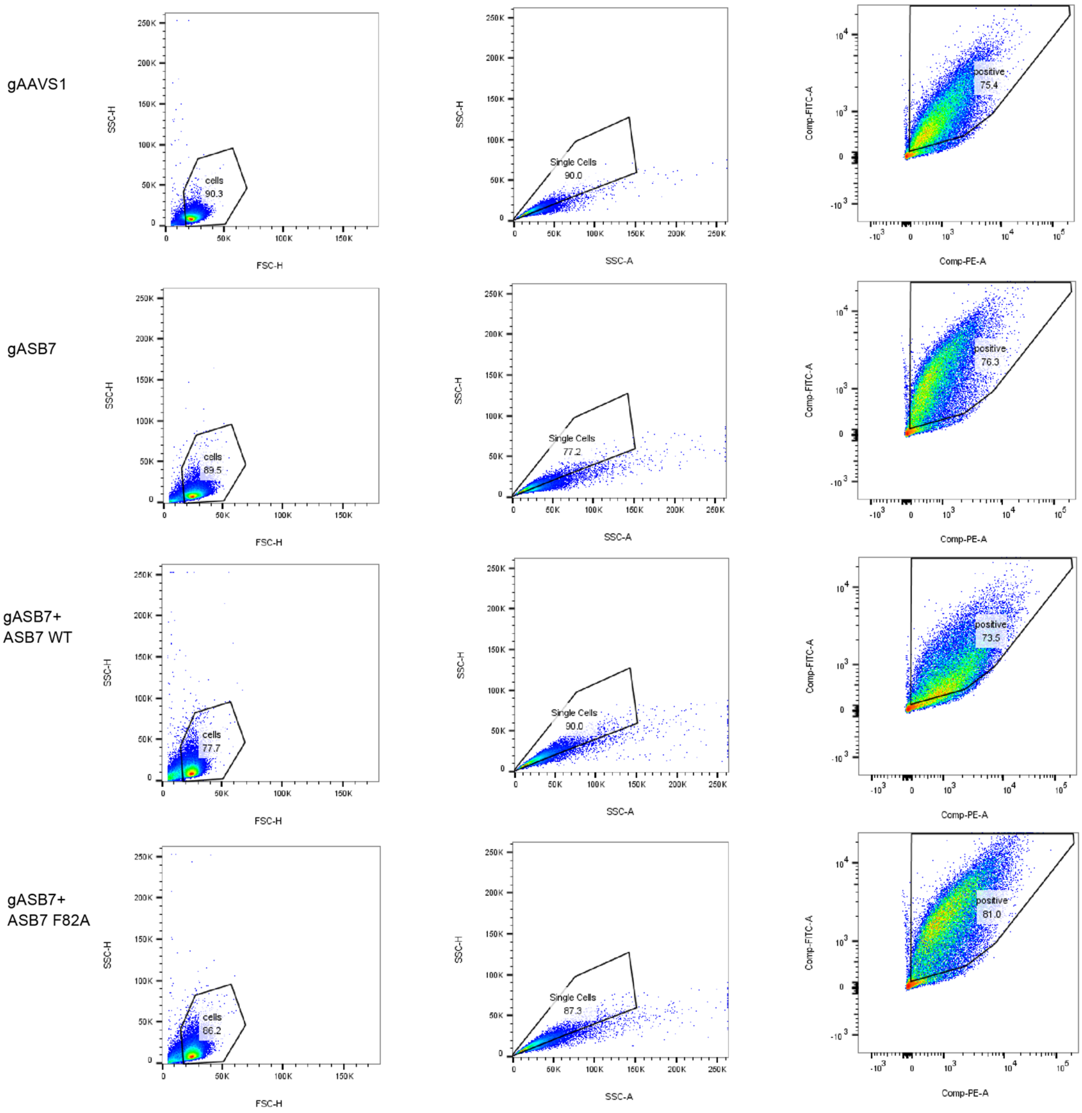

For Fig.5
CEP152

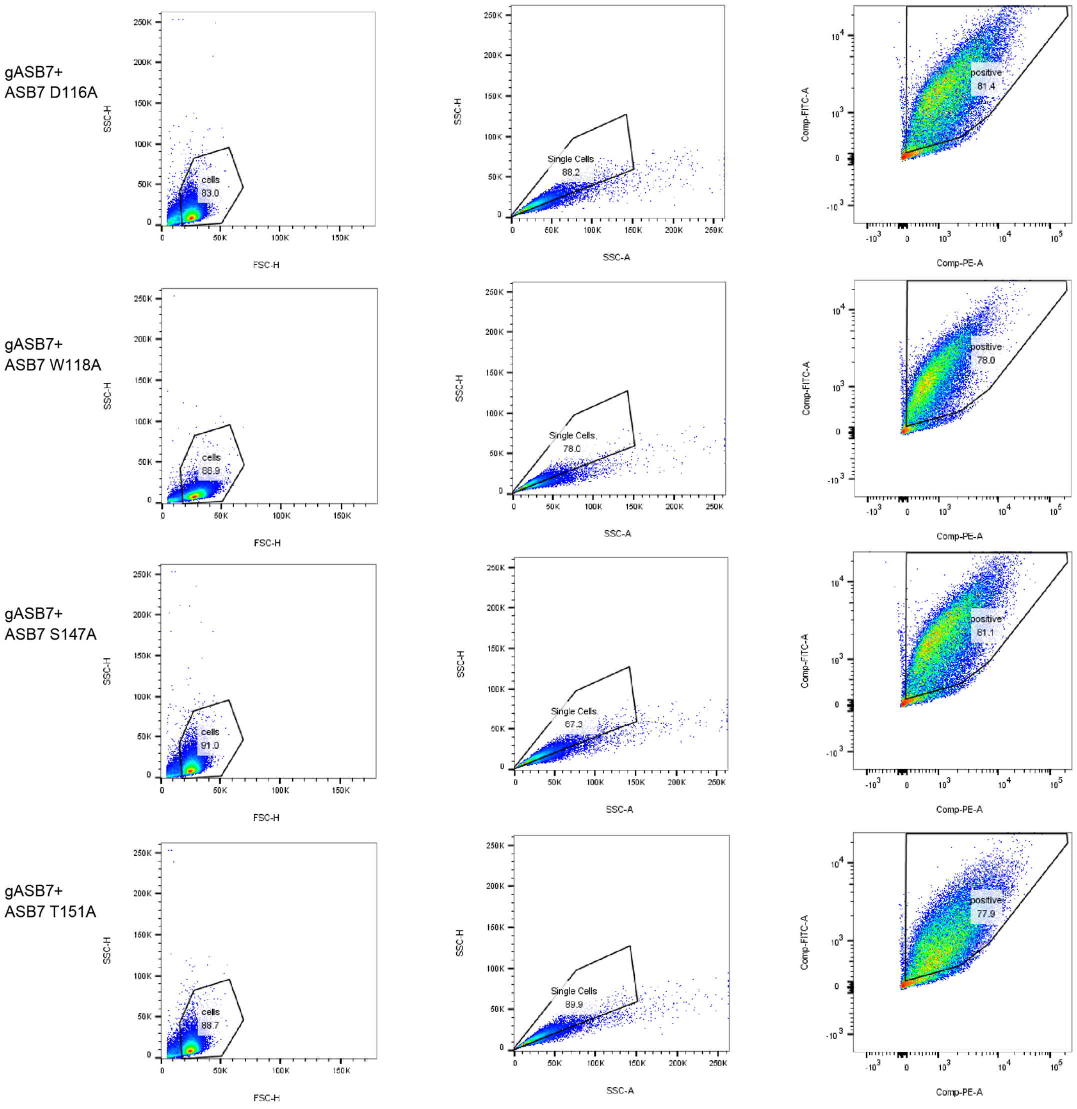

For Supplementary Fig. 2
DDA3

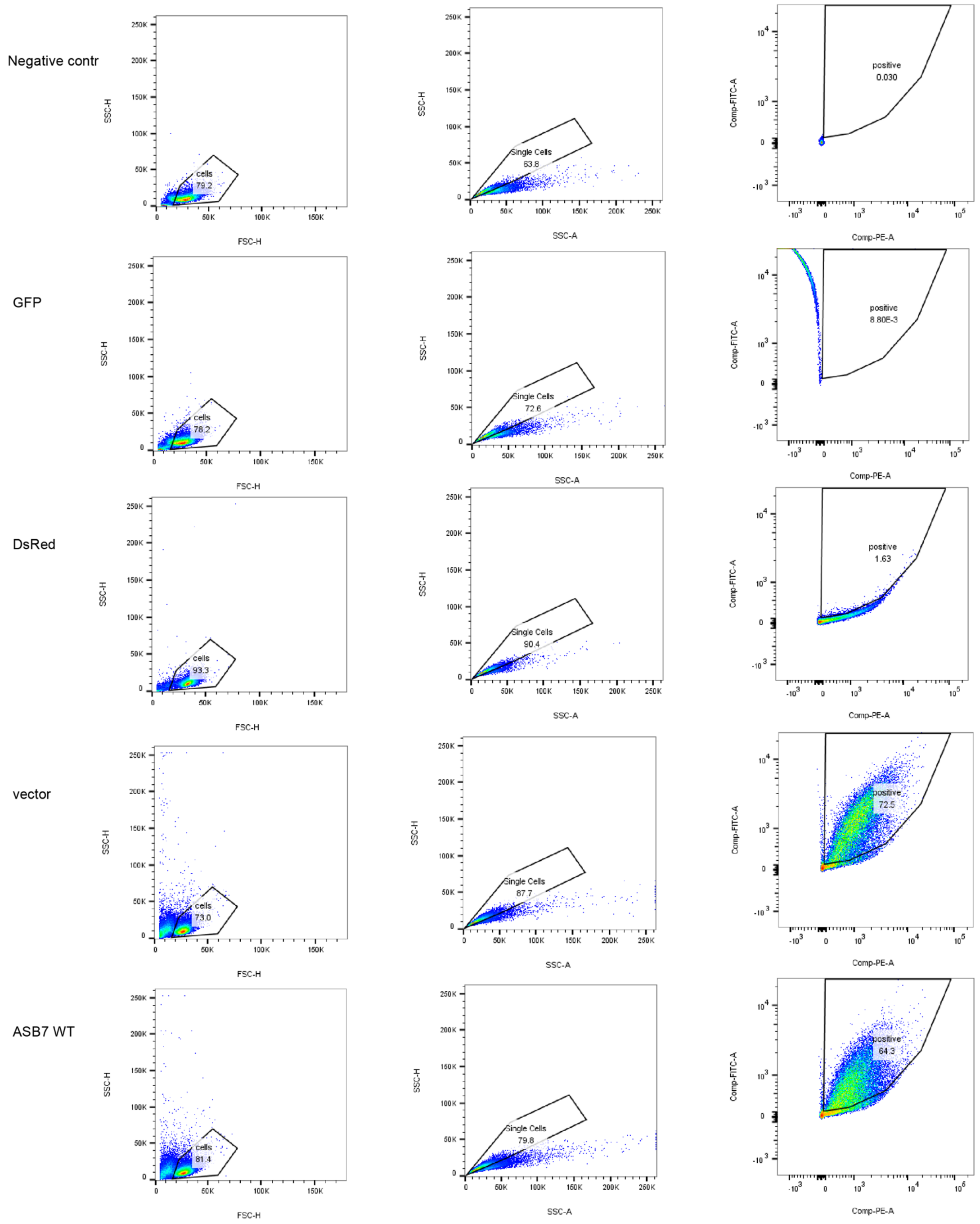

For Supplementary Fig. 3
LZTS1

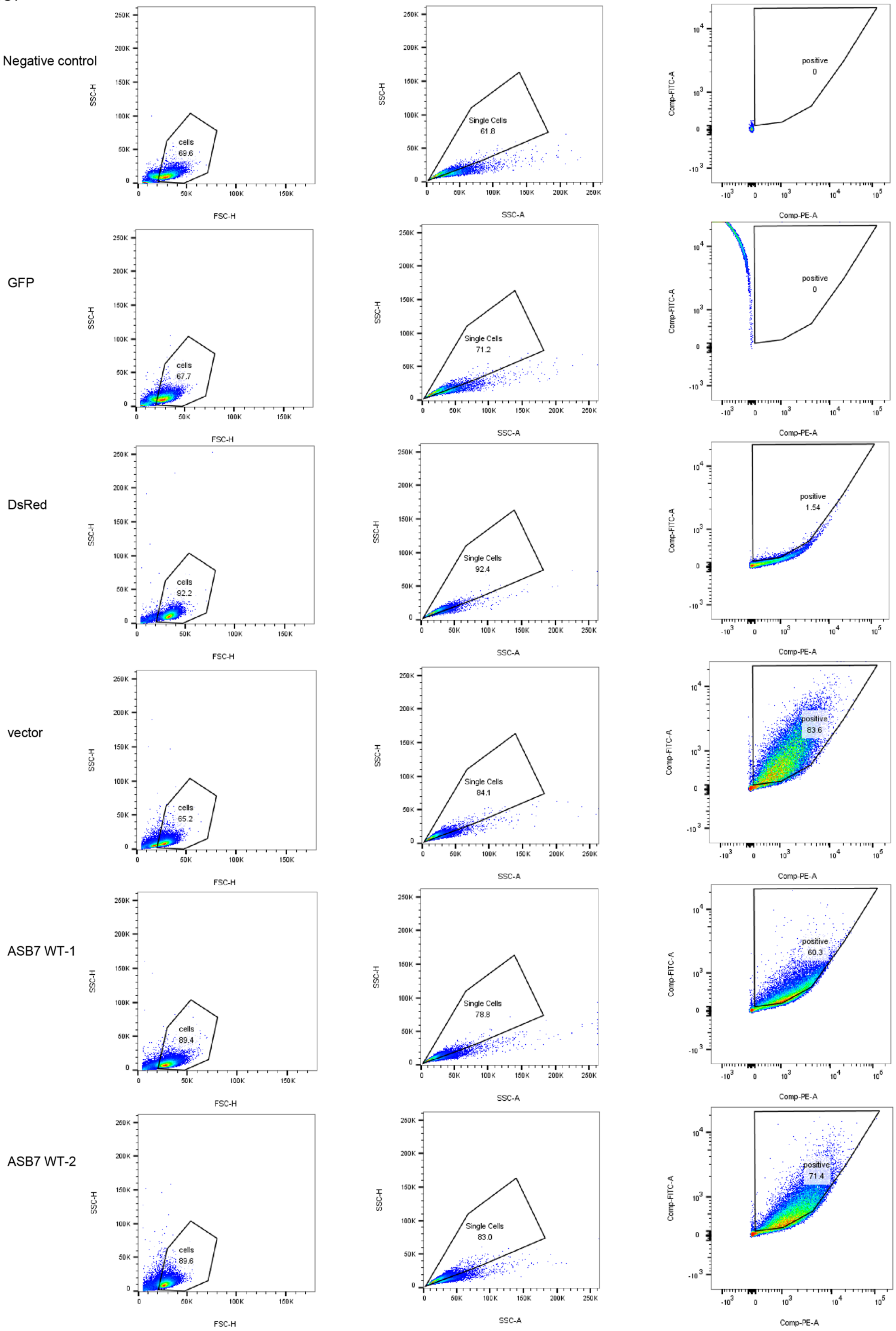

For Supplementary Fig. 3
LZTS1

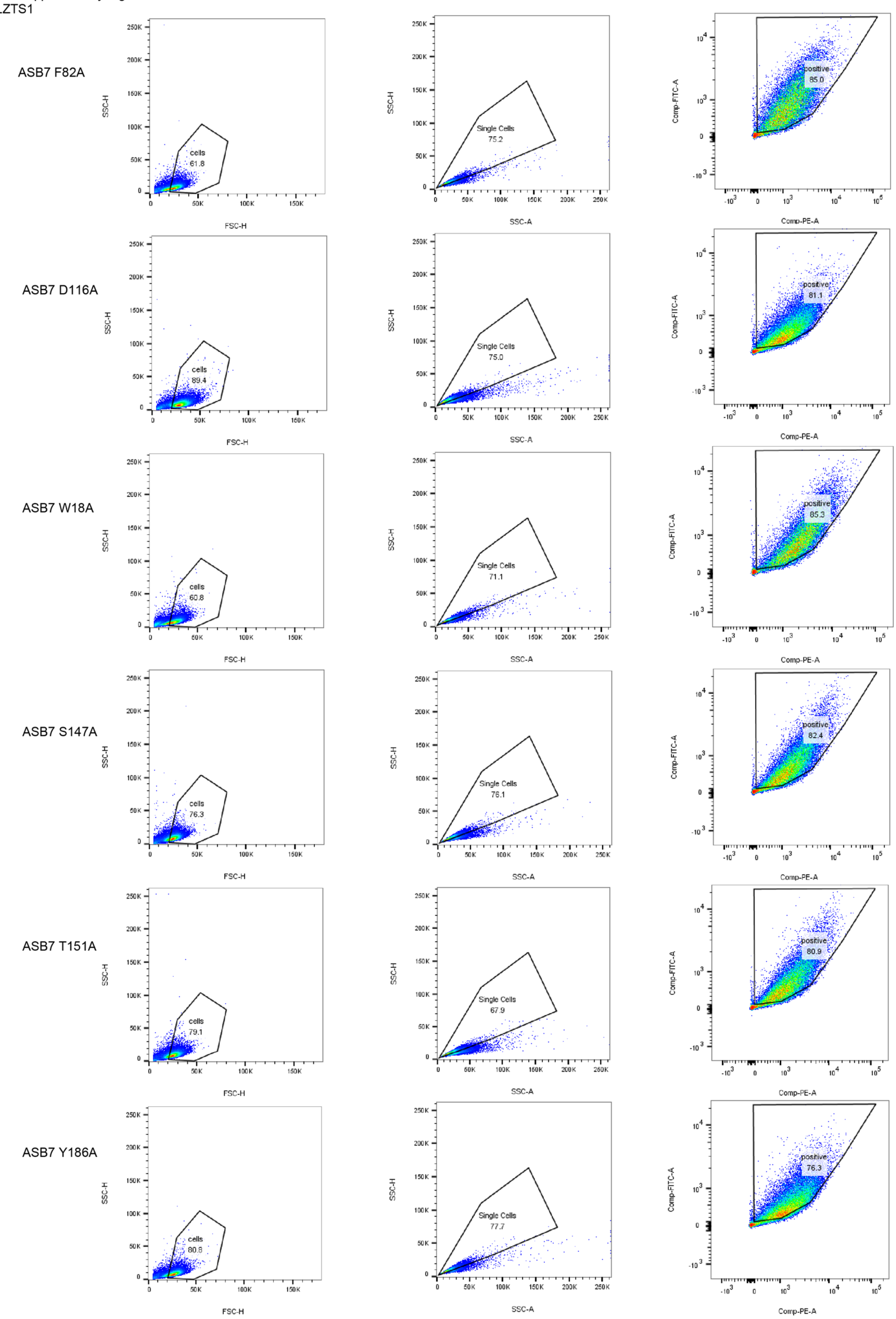

For Supplementary Fig. 3
CEP52

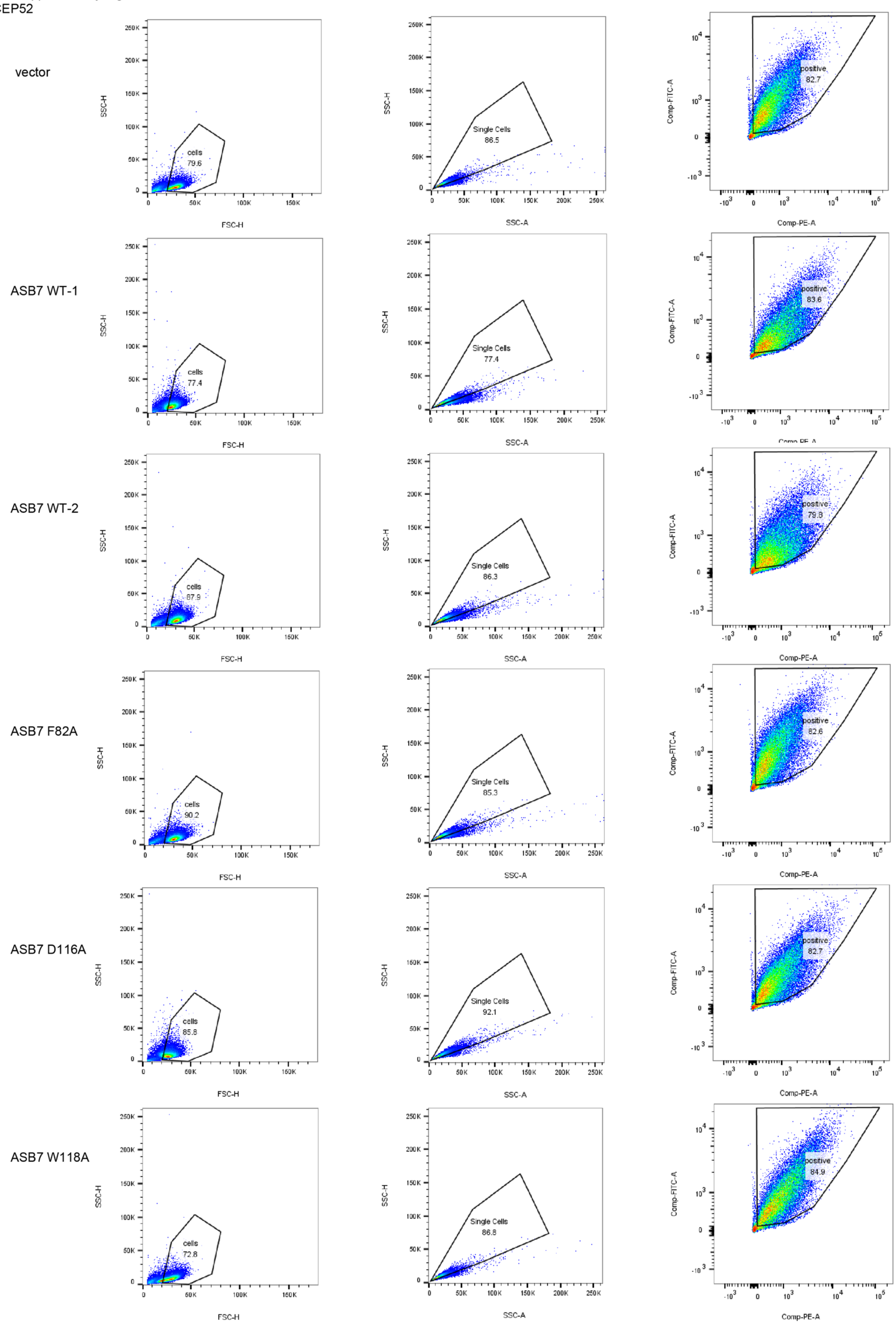

For Supplementary Fig. 3
CEP52

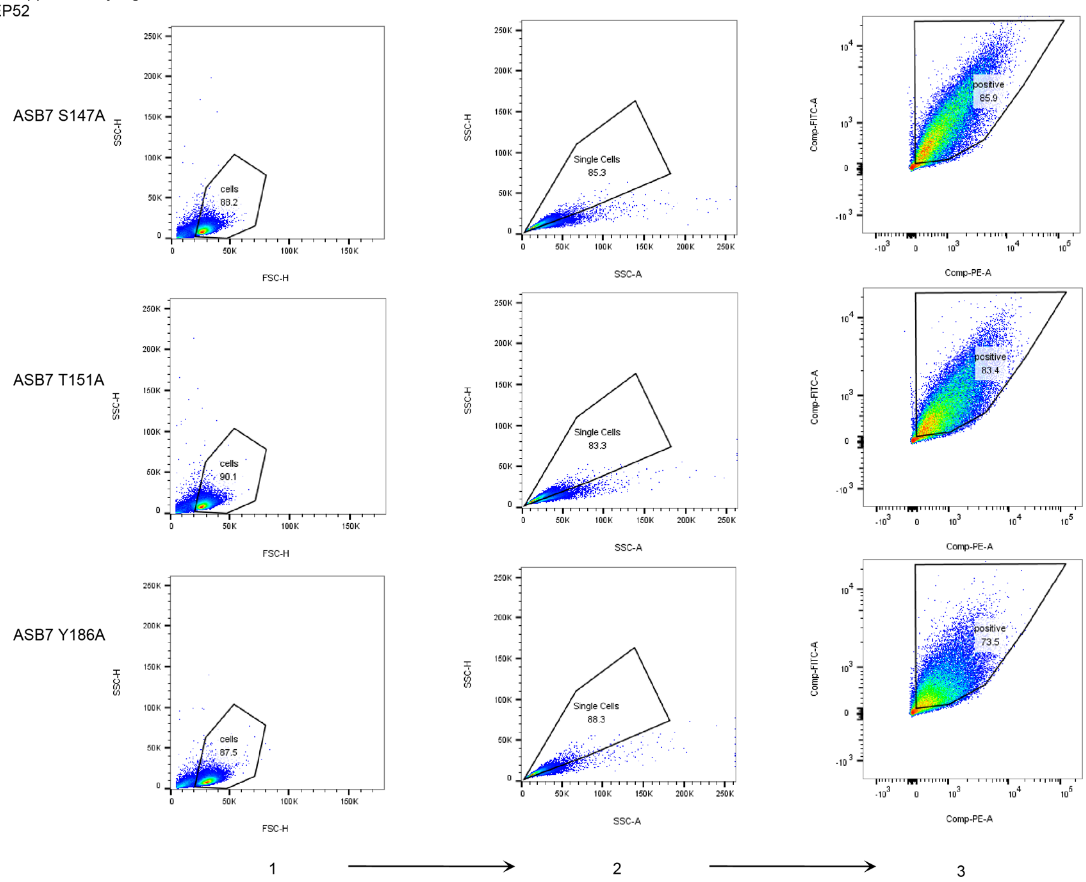

Supplementary Fig. 5 | Gating images for FACS. 1. Gate on FSC-H vs. SSC-H was set to include all cell populations, but excluding debris; 2. Gate on SSC-A vs. SSC-H was set to exclude doublets; 3. The selected cells was calculated for GFP/DsRed ratio.

Supplementary Table 1 | Thermodynamic parameters of ITC measurement of various peptides and ASB7-Elongin B/C.

**peptide in syringe**	**protein in cell**	K_D **(μM)**	**ΔH (kcal/mol)**	**ΔG (kcal/mol)**	**-TΔS (kcal/mol)**	**N**
**EDDEGLKHLQQEAEKLVASLQDS (GIGYF1)**	WT	1.0±0.41	-4.54±0.32	-7.91	-3.37	0.78±0.02
**LEQEIRELQAEAGRTRGALEVLG (CCDC17)**	WT	1.1±0.39	-14±0.84	-8	5.95	0.76±0.02
**ELEKAKQECQDLKGKLEKCCRH (CEP152)**	WT	3.8±0.45	-1.8±0.09	-6.59	-4.79	1.27±0.03
**EIGELKAVMEGIKMEHQLELGN (CLIP2)**	WT	0.7±0.12	-10.8±0.25	-8.23	2.57	0.71±0.01
**RQELESLMKEQDLLETKLRSYER (LZTS1)**	WT	0.8±0.06	-7.58±0.08	-8.09	-5.04	0.99±0.01
**PEKLEEILDEANRLAAQLEQCAL (DDA3)**	WT	1.3±0.44	-2.57±0.13	-7.77	-5.19	1.32±0.03
**RQERESLMKEQDLLETKLRSYER**	WT	8.2±1.24	-4.81±0.49	-6.53	-1.73	1.03±0.04
**RQELESRMKEQDLLETKLRSYER**	WT	NB				
**RQELESLMKRQDLLETKLRSYER**	WT	NB				
**RQELESLMKEQDLRETKLRSYER**	WT	NB				
**RQELESLMKEQDLLETKRRSYER**	WT	NB				
**RQELESLMKEQDLLETKLRSRER**	WT	0.7±0.05	-6.71±0.07	-8.2	-1.5	0.71±0.01
**RQELESLMKEQDLLETKLRSYE**	WT	0.6±0.36	-4.6±0.34	-8.28	-3.68	0.90±0.03
**RQELESLMKEQDLLETKLRSY**	WT	0.9±0.16	-5.39±0.13	-7.96	-2.57	0.91±0.01
**QELESLMKEODLLETKLRSYER**	WT	0.6±0.15	-4.53±0.14	-8.27	-3.74	0.75±0.01
**ELESLMKEQDLLETKLRSYER**	WT	0.7±0.31	-7.49±0.52	-8.25	-0.75	0.68±0.02
**ELESLMKEQDLLETKLR**	WT	5.1±2.2	-3.33±0.35	-6.8	-3.46	1.12±0.03
**ELESLMKEQDLLE**	WT	NB				
**ELESLMKEQ**	WT	NB				
**ELESLM**	WT	NB				
**RQELESLMKEQDLLETKLRSYER**	F82A	8.7±1.4	-4.46±0.36	-6.7	-1.42	0.79±0.03
**RQELESLMKEQDLLETKLRSYER**	F82D	NB				
**RQELESLMKEQDLLETKLRSYER**	W118A	21±3.7	-3.45±0.31	-6.28	-2.83	1.44±0.05
**RQELESLMKEQDLLETKLRSYER**	W118D	NB				
**RQELESLMKEQDLLETKLRSYER**	S147A	30±9.6	-2.54±0.61	-5.97	1.36	0.66±0.12
**RQELESLMKEQDLLETKLRSYER**	T151A	14±3.6	-1.14±0.12	-6.58	-5.43	1.12±0.09
**RQELESLMKEQDLLETKLRSYER**	F182A	2.4±0.88	-3.2±0.23	-7.4	-4.2	1.01±0.04
**RQELESLMKEQDLLETKLRSYER**	R185A	3.1±0.48	-3±0.11	-7.34	-4.34	0.84±0.02
**RQELESLMKEQDLLETKLRSYER**	Y87A	0.8±0.06	-5.67±0.21	-8.79	-3.12	1.23±0.02
**RQELESLMKEQDLLETKLRSYER**	D116A	5.4±0.85	-4.4±0.17	-6.96	-2.56	1.24±0.02
**RQELESLMKEQDLLETKLRSYER**	H126A	0.7±0.12	-6±0.15	-8.26	-2.26	0.77±0.01
**RQELESLMKEQDLLETKLRSYER**	Y127A	0.7±0.1	-5.42±0.08	-8.12	-2.71	1.48±0.01
**RQELESLMKEQDLLETKLRSYER**	R160A	2.2±0.32	-5.01±0.13	-7.43	-2.41	0.90±0.01

**RQELESLMKEQDLLETKLRSYER**	Y186A	1.5±0.82	-7.35±1.76	-6.95	0.4	1.03±0.06
**RQELESLMKEQDLLETKLRSYER**	I189A	0.7±0.08	-5.38±0.08	-8.19	-2.81	1.10±0.01

The ITC assay was independently repeated three times. Values reported are the mean ± SD.
